# Mining Association Rules From a Multimodal Dataset of a Digital Therapeutics Application for Sleep Improvement Through a Healthy Lifestyle: Quantitative Study

**DOI:** 10.2196/75358

**Published:** 2026-07-06

**Authors:** Luka Biedebach, Katrín Ýr Friðgeirsdóttir, Camilla Carpinelli, Ari Páll Isberg, Halla Helgadóttir, Erna Sif Arnardóttir, Jose M Saavedra, Anna Sigridur Islind

**Affiliations:** 1 Department of Computer Science, Reykjavík University, Menntavegur 1, Reykjavik, 101, Iceland, 354 5996200; 2 Reykjavik University Sleep Institute, Reykjavík University, Reykjavik, Iceland; 3 Physical Activity, Physical Education, Sport and Health (PAPESH) Research Centre, Department of Sport Science, Reykjavík University, Reykjavik, Iceland; 4 Sidekick Health, Reykjavik, Iceland; 5 Department of Engineering, Reykjavík University, Reykjavik, Iceland

**Keywords:** sleep, digital therapeutics, DTx, wearables, association rules, multimodal dataset

## Abstract

**Background:**

The demand for sleep interventions is high and steadily growing. Digital therapeutics (DTx) can help individuals improve their sleep remotely, over an extended period, and with less effort from medical professionals. Obstructive sleep apnea (OSA), one of the most prevalent and consequential sleep disorders, can be treated with health-supporting behavior changes, such as physical exercise and weight loss, and, therefore, acts as a promising application for DTx.

**Objective:**

The study aimed to analyze a digital intervention from both medical and technological perspectives by moving beyond clinical markers and exploring more deeply how the DTx application was used. This study aimed to propose a novel way in which association rules can function as an exploratory tool to analyze the sleep, behavior, and engagement of participants with the DTx application on a day-to-day level.

**Methods:**

A lifestyle intervention study (N=192) targeted at adults with mild-to-moderate OSA aimed to reduce their OSA severity using a DTx application and an exercise program over a study period of 12 weeks. The participants’ OSA severity was assessed through polysomnography at the beginning and at the end of the study period, and the participants tracked their sleep with a digital sleep diary and a smartwatch over the course of the entire study. The DTx application provided data on when and how the participants pursued the proposed lifestyle interventions. These heterogeneous data sources were combined into one multimodal dataset, which was explored through descriptive statistics. Ultimately, the data were turned into a transaction-based format, and association rules were derived using the Apriori algorithm.

**Results:**

Analyzing the participants’ interaction with the application revealed the lifestyle interventions they pursued and how their behavior and sleep patterns changed over time. The Apriori algorithm generated a set of association rules with lift and confidence scores that were significantly higher than those for the co-occurrence of items through random chance. The rules show co-occurrence of missions and items from the sleep diary, as well as items derived from the watch measurements.

**Conclusions:**

The study showed the richness of the various data sources provided by a digital intervention using wearables and how they can be used to get an in-depth understanding of the study. The generated association rules showed the presence of significant co-occurrences across the different data modalities and highlighted their effectiveness as an exploratory tool for multimodal health data.

## Introduction

### Background

Digital therapeutics (DTx) are expected to influence the way health care is delivered all around the world [1]. Wang et al [2] define DTx as “software that provides evidence-based medical interventions for disease or disorder prevention, management, and treatment,” which makes DTx a central element within the research field of digital health. This 3-fold focus on preventing, managing, and treating diseases through DTx has been shown to be successful for chronic and difficult-to-treat diseases and to lead to sustainable long-term outcomes [3]. Since sleep is one of the pillars of health [4], and obstructive sleep apnea (OSA) is highly prevalent in the general population [5], it is vital to study the relationship between DTx and OSA treatment. DTx has the strength of delivering care remotely, which enables users to take part in interventions over a continuous and extended period in the comfort of their own homes. It is a “one-to-many model of care,” providing personalized health care while using fewer resources [6]. Fürstenau et al [7] identified the combination with wearables, which can track sleep and activity longitudinally, as a promising future research area. In this work, we will analyze usage patterns in the DTx application enriched with information from a digital sleep diary and clinical markers from a lifestyle study for adults with OSA.

**Figure 1.**
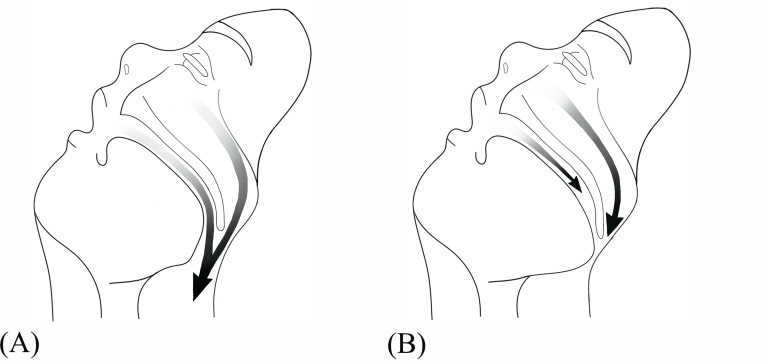
(A) Normal breath versus (B) an upper airway obstruction.

Sleep-disordered breathing encompasses a range of breathing-related sleep disorders, from habitual snoring to severe OSA. OSA is the most common form of sleep disorder worldwide and is characterized by temporary interruptions of breathing during sleep due to upper airway obstruction [8,9]. Globally, an estimated 425 million individuals experience moderate-to-severe OSA (defined as 15 or more events per hour) [10]. Clinically, apnea is defined as a complete cessation of airflow lasting more than 10 seconds, while hypopnea refers to a partial reduction of airflow (by at least 30%) for at least 10 seconds [11]. The Apnea-Hypopnea Index (AHI) quantifies these events per hour [5]. These disruptions frequently lead to reduced blood oxygen levels, triggering awakenings from sleep, which, in turn, prevent patients with OSA from achieving sufficient restorative sleep. As they often return to normal breathing without recalling the interruptions, approximately 80% of individuals with OSA remain undiagnosed [12]. Figure 1 illustrates the mechanism of airway obstruction in patients with OSA.

The most common treatment for OSA is continuous positive airway pressure, which is an effective way to eliminate breathing events. However, patients often struggle with treatment adherence due to discomfort, inconvenience, or a lack of perceived benefit. Contemporary research aims to improve outcomes for individuals with OSA without relying on medical devices but rather by initiating health-supporting behavior changes, for example, an exercise-based lifestyle intervention [13-15], reducing obesity levels [16,17], or improving muscle tone with myofunctional therapy [18]. In this context, digitally delivered interventions for OSA are a promising research direction to educate patients, promote healthy behavior change, and remotely monitor progress over an extended period.

DTx represents a convergence of medical science, interaction design, and computer science, with the aim of transforming health care in general and chronic disease management in particular. Therefore, when testing their effectiveness on participants, the application should be viewed from different perspectives, including known clinical markers of treatment success as well as insights from user engagement. In this paper, we focused our efforts on answering the following research questions (RQs):

- RQ1: What are the engagement patterns of participants in the DTx application?
- RQ2: How is engagement related to changes in the participant’s behavior and sleep?

In the following sections, we review existing research on DTx for sleep improvement and DTx using wearables, introduce the lifestyle intervention study, describe the statistical methods used for analysis, and discuss the results in the context of existing literature on digital health and personalized medicine.

### Related Work

Behavioral interventions are a common approach to improving sleep quality for people with and without sleep disorders [19]. A web-based DTx application to improve sleep efficiency showed significant improvements for people with insomnia [13]. Web-based DTx for sleep improvement has also shown positive effects on the sleep of people without insomnia [20]. Comparing different forms of DTx, Luik et al [21] identified three levels: (1) DTx as support for offline therapy, (2) DTx as the main therapy with the support of a health professional, and (3) DTx as a fully automated digital intervention program. One example is the work by Wickwire et al [22], who used a combination of web- and app-based DTx in parallel with a commercial off-the-shelf sleep tracker. There are no publications focusing on DTx applications for OSA treatment in the existing literature, making this research area highly relevant. Fridgeirsdottir et al [16] conducted a randomized controlled trial to use exercise and a DTx application to reduce OSA severity. We aimed to build upon and complement their work with an in-depth analysis of the DTx intervention group.

Research has extensively demonstrated the effectiveness of digital interventions for sleep improvement, but most research has been conducted in the medical field. However, given the technological nature of these interventions, a comprehensive evaluation requires considering both medical and digital perspectives. Specifically, the interaction of the participant with the application is crucial for ensuring that such interventions are engaging, intuitive, and tailored to their needs. Aji et al [23] conducted a systematic review of existing DTx applications for sleep improvement. They identified personalized sleep feedback, educational material, and a digital sleep diary as the most common features. Understanding how participants engage with digital health interventions and whether that engagement translates into better outcomes is an active area of research in medicine and health informatics. Previous studies have approached this issue by analyzing engagement data to identify usage metrics or patterns and then examining their association with clinical or behavior outcomes [24,25]. Their findings suggest that patients who actively use and participate in digital interventions are more likely to benefit from them. Nelson et al [26] propose different strategies to assess the relationship between engagement and health outcomes. Gan et al [27] conducted a literature review and meta-analysis in 2021, which showed that engagement with digital mental health interventions is associated with higher health outcomes.

Association rule mining is used to analyze co-occurrences of events and has been applied in different fields within digital health. For example, Concaro et al [28] used clinical variables from medical health records to analyze the relationship between clinical variables and drug effects. There are multiple publications that have used association rule mining to identify patterns in the data from sleep questionnaires, sleep parameters from polysomnography (PSG), and demographic information about participants [29-31]. Altaf et al [32] discussed 35 studies in their survey from 2016 that used association rules in the context of health informatics. The publication by Liang et al [33] shows how longitudinal data from a smartwatch can be used to discover factors relevant to people’s sleep. We pursue a similar research direction by applying association rule mining on the data collected through a DTx application, identifying patterns in participant engagement with the sleep lifestyle intervention. Alslaity et al [34] used association rules to analyze the interaction of users with a mental health app. Our work aims to take this research approach one step further by integrating app engagement data with other types of measurement data.

## Methods

### Study Context

This research is a secondary analysis based on a randomized controlled trial registered in the ISRCTN Registry (ISRCT16974764), which was conducted as part of the European Union Horizon Project Sleep Revolution [35]. The study spanned a 12-week period, during which participants were asked to (1) complete a 3-night self-applied PSG at the beginning and end of the study; (2) wear a Withings ScanWatch; (3) report their sleep using a digital sleep diary mobile app; and (4) follow a lifestyle intervention program aimed at improving their lifestyle and, ultimately, their OSA over the 12 weeks [14]. The study population included 192 adults aged between 18 and 50 years with a BMI between 25 and 42 kg/m^2^. The gender balance was 50.9% (98/192) male individuals. The participants had mild-to-moderate OSA, with an AHI between 5 and 30. The study population was randomized into 3 intervention groups. The A* Pathfinding algorithm was used to stratify the groups by age, sex, BMI, and AHI. Only 1 of these groups used a digital health program developed by Sidekick Health, which is the focus of the analysis in this paper. The participants used the Sidekick Digital Therapeutics application as the intervention program. Fridgeirsdottir et al [16] conducted a statistical analysis on all 3 groups of the randomized controlled trial. Their findings showed that the DTx group experienced reductions in weight, neck circumference, body fat, visceral adiposity, and skeletal muscle mass. However, there was no significant improvement in OSA severity within the DTx group. In this paper, we aim to extend their work by analyzing the participants’ engagement with the DTx application, as well as changes in their sleep and behavior on a day-to-day basis. The DTx intervention group included 55 participants with a mean age of 37.1 (SD 6.8) years and a mean BMI of 32.9 (SD 4.1) kg/m^2^. They had an average AHI of 12.1 (SD 9.7), that is, on average, 12 apneas or hypopneas occurred per hour of sleep. An overview of the demographic information for the DTx group can be found in Table 1.

The DTx application provides the participant with suggestions for behavioral interventions to improve their sleep. The DTx application guides participants to improve their lifestyle by goal setting, self-monitoring, and completing health-related tasks. This touches on different areas of a healthy lifestyle: (1) creating awareness of eating habits, nutrition, and diet through educational material and meal logging; (2) attempting to prevent stress through relaxation, meditation, and mindfulness exercises; (3) motivating participants to engage in physical activity and exercise; (4) providing general health education to participants; and (5) helping participants take care of their own health through tracking and reminders. The overall goal of the intervention is to increase the frequency of healthy lifestyle behaviors and, by doing so, improve the health of participants.

**Table 1.** Demographic information of the analyzed study population.

Variable	Mean (SD)
Age (y)	37.1 (6.8)
BMI (kg/m^2^)	32.9 (4.1)
Apnea-Hypopnea Index (events/h)	12.1 (9.7)

The intervention concept of the application is based on *missions* that the participant is asked to follow. Missions addressing different areas of a healthy lifestyle appear on the dashboard, and participants can select which ones they want to complete. An overview of the different mission categories can be seen in Table 2. The types and content of the missions have been designed particularly for sleep improvement. However, the application can be customized for other conditions and interventions as well and has shown success, for example, in obesity [36] and diabetes [37]. The application uses gamification in terms of design, competition, and rewards. In this way, the DTx application uses storytelling to frame the missions as enjoyable tasks. The tasks provide instant gratification to participants by showing personal achievements and fostering social interactions. Participants track their personal achievements by collecting points, which earn them virtual rewards. Social interaction is created through messages from a coach, with whom participants can communicate through the app. Additionally, a mascot is included to motivate participants to follow the interventions. These gamification elements aim to nudge participants toward healthier behavior [38].

**Table 2.** Types of missions.

Mission	Description
Education	Video, audio, and written content are shown to participants. The educational material is tailored to the specific intervention program and includes content on good habits to improve sleep, as well as general lifestyle and health education.
Clinic	Involves logging weight, blood pressure, and other physical measurements, as well as setting reminders for taking supplements.
Mind	Participants are asked to log their stress level, energy levels, as well as their quality of sleep. This category furthermore involves different breathing and meditation exercises.
Move	Participants record their physical activity, which may range from daily steps to structured exercise or sports.
Food	Food intake is tracked and categorized into vegetables, nuts, fruit, snacks, and candy. Beverage intake is tracked and categorized into water and soda. This category also includes alcohol and nicotine.

### Ethical Considerations

The study is covered by the ethical approval of the National Bioethics Committee of Iceland (VSN-22‐082). All participants were informed about the purpose of this study, and written consent was a prerequisite for participation. Data are securely stored on a local machine in accordance with data protection standards. In the presented data, individuals are no longer identifiable, either directly or indirectly.

### Data

The study setup, which includes PSG, a smartwatch, a digital sleep diary, and the DTx app, results in a diverse dataset. In the following sections, we are going to explain our 4 main data sources, provide an overview of the size of each dataset, and describe the features that are included.

#### PSG Data

The PSG data were manually reviewed by a sleep expert according to the American Academy of Sleep Medicine scoring manual [39]. The self-applied PSG setup included the classical sensors, with simplified electroencephalography using only frontal electrodes [40]. The participants recorded 1 to 3 nights of sleep with this setup before and after the intervention period. The AHI values derived from the manual scoring of these recordings (up to 3 nights) were averaged to one value before and one value after for each participant.

#### DTx Data

The data from the DTx application is organized by the missions that participants completed. Each mission record contains information about the specific mission name, mission category, and the completion day and time. In total, there were 92 different missions across 5 mission categories. Participants could complete multiple missions per day. Apart from the mission dataset, we also had information about how often the participant interacted with a health coach through the app. Here, only the day of interaction and whether the participant sent or received a message were recorded. The content of the messages was not recorded.

#### Digital Sleep Diary Data

As part of the study, the participants used a digital sleep diary app. It was developed within the Sleep Revolution project and is based on the research of Schmitz et al [41]. In the app, the participants were asked to report the quality of their sleep in the morning and report information about their day in the evening. The subjective sleep quality and stress levels were reported on a Likert scale, with 1 being the lowest and 5 the highest rating. Participants also reported how long they stayed in bed awake in the morning before getting out of bed. The participants had an average sleep quality rating of 2.14 (SD 0.37). In the evening diary, the participants were asked about their stress levels, pain, screen time, exercise, and medication intake.

If the participants did not fill out the sleep diary, they were reminded through notifications. The participants filled out the morning diary for an average of 48 (SD 29.35) days and the evening diary for an average of 42 (SD 26.23) days. We merged the diary entries from the evening diary with the entries from the morning diary on the following day. Therefore, each record in the dataset represents 1 night of sleep.

#### Smartwatch Data

The Withings ScanWatch has been validated for sleep tracking [42] and reports various sleep parameters, including sleep duration, the time participants spent awake during the night, and the number of nocturnal awakenings. These awakenings could range from seconds to several minutes or hours. The smartwatch also tracked sleep onset latency, defined as the time taken to fall asleep. Beyond sleep, the smartwatch also recorded the number of steps the participants took during the day. The participants were asked to wear the smartwatch for the entire study duration, but there were varying levels of adherence. On average, each participant wore the smartwatch for 62 days over the entire study period of 12 weeks. The average sleep duration across participants was 7 hours and 33 minutes, with an SD of 55 minutes.

### Data Analysis: Association Rule Mining

As a first step, the health data from these different sources were merged, joining the records by participant identifier and time stamp. Descriptive data analysis was then used to provide an initial understanding of the participants’ behavior over time and their adherence to the intervention program.

To analyze the co-occurrence of lifestyle interventions, we applied association rule mining. Association rule mining is a machine learning technique used to uncover relationships between items in a dataset based on their co-occurrence [43]. Association rule mining is an unsupervised machine learning method commonly used to identify patterns, such as items that customers buy together or pages of a website that users visit together.

Frequently co-occurring items, known as frequent item sets, are used to identify rules with an “if-then” structure; for example, if item *X* occurs, then item *Y* is likely to occur. In such rules, *X* is referred to as the antecedent, and *Y* is the consequent. These rules rely on three key metrics: support, confidence, and lift. Support determines the frequency with which items co-occur. If we notate a transaction as *N* and an item as *x*, we can define support as the following equation:


(1) $$support(x_{1}\to x_{2})=\frac{N(x_{1}\cup x_{2})}{N}$$

where *N* is the total number of sets, and *N*(*x*_1_ ∪ *x*_2_) is the number of sets containing both items *x*_1_ and *x*_2_. Confidence represents the likelihood of the consequent given the antecedent, where *N*(*x*_1_) is the number of transactions containing *x*_1_:


(2) $$confidence(x_{1}\to x_{2})=\frac{N(x_{1}\cup x_{2})}{N(x_{1})}$$

Lift assesses the strength of an association by comparing the observed co-occurrence of items to the probability of their co-occurrence if they were statistically independent:


(3) $$lift(x_{1}\to x_{2})=\frac{confidence(x_{1}\to x_{2})}{support(x_{2})}=\frac{N(x_{1}\cup x_{2})\cdot N}{N(x_{1})\cdot N(x_{2})}$$

The most common algorithm for generating association rules is the Apriori algorithm [44]. It identifies frequent item sets by calculating support for all item combinations and retaining those that meet a minimum support threshold. These sets are expanded iteratively until no new frequent item sets are found. Rules are then derived by calculating confidence for each potential rule, filtering those that meet a minimum confidence threshold, and using lift to eliminate coincidental associations. Since association rules work with categorical data, all numerical columns had to be binned. The binning was manually decided based on the distribution of each feature. Table 3 shows how the sleep parameters were binned.

**Table 3.** Binning of numerical features.

Feature	Bins↑	Bins↓
Sleep duration (h)	>8	<6
Awakenings (count)	>5	0
Sleep onset latency (min)	>60	<10
Awake (h)	>1	—^a^
Screen time (h)	>8	<2
Sleep quality (Likert scale, 1=“lowest” to 5=“highest”)	≥4	≤1
Staying awake in bed (min)	>60	<15
Activity (steps)	>10,000	<1000
Stress (Likert scale, 1=“lowest” to 5=“highest”)	≥4	≤1

a Not available.

The dataset was transformed into a transaction-based format, where each transaction referred to 1 night of sleep by 1 individual. The items in the transaction were the categorical features derived from the smartwatch and the DTx application. Merging the data from the smartwatch, the digital sleep diary, and the DTx application led to a transaction-based dataset with 3642 transactions. In the following, an exemplary transaction of participant *n* on day *m* shows a decreased sleep duration, a completed education mission (*M*_Education_), and increased awakenings:


(4) $$X_{n}Y_{m}=Duration↓,M_{Education},Awakenings↑$$

The uniqueness of association rules lies in analyzing the data in the form of transactions and, therefore, handling complex datasets, including multiple features of multiple participants on multiple nights. This allows us to analyze sleep, behavior, and engagement on a day-by-day level in this study. Given that participants could choose different missions based on their individual preferences, association rule mining enables the identification of frequent combinations of interventions used and related changes in sleep and behavior. The chi-square test was used to validate the significance of the rules as proposed by Brin et al [45]. It validates the null hypothesis that the occurrence of item set *X* is independent of the occurrence of item set *Y* in the dataset. The expected occurrence is compared to the observed occurrence of *X* and *Y*, both individually and together. Finally, this test results in a chi-square value and a *P* value, which describe the strength of the association:


(5) $$\chi^{2}=\sum_{i}\frac{(O_{i}-E_{i}{)}^{2}}{E_{i}}$$

To further demonstrate the significance of our rules compared to random chance, we conducted a permutation test, which compares the generated rules to 1000 sets of rules derived from randomly generated data. We created 1000 synthetic datasets, all of which have the same frequency of items but are randomly shuffled through the transactions. In this way, we show a dataset where all items are independent of each other. This approach allows the evaluation metrics—support, confidence, and lift—to be validated by comparing them to random chance. The code for the frequent item set and rule generation, as well as the evaluation alongside a synthetically created dataset, can be found in Multimedia Appendix 1.

## Results

### Overview

In the following section, we will explain in detail how and how often participants engaged with the DTx application. Dividing the data by the changes in AHI further showed differences in engagement with the application. Finally, based on the different intervention types, frequent patterns and association rules were derived.

**Figure 2.**
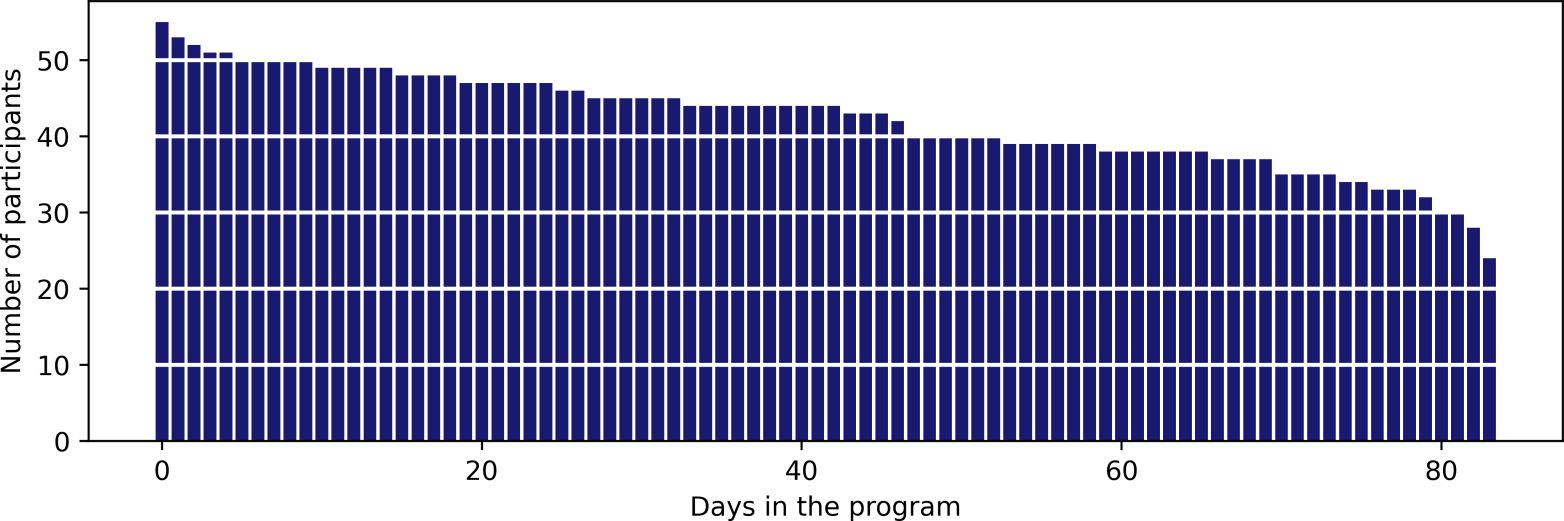
Participant retention in the app throughout the 12-week study duration.

The first step in gaining insights into the effectiveness of DTx for improving sleep quality was collecting information about the 12-week study period. We analyzed participant retention by identifying how many individuals completed the study, along with their levels of engagement with the DTx application throughout the intervention period. This included an analysis of the frequency and consistency with which participants interacted with the app. Even though there was a dropout of participants over time, 43 participants adhered to the study design for more than half of the study period. Furthermore, 30 of the 51 participants used the application for more than 80 days. Figure 2 shows the dropout of participants over time.

**Figure 3.**
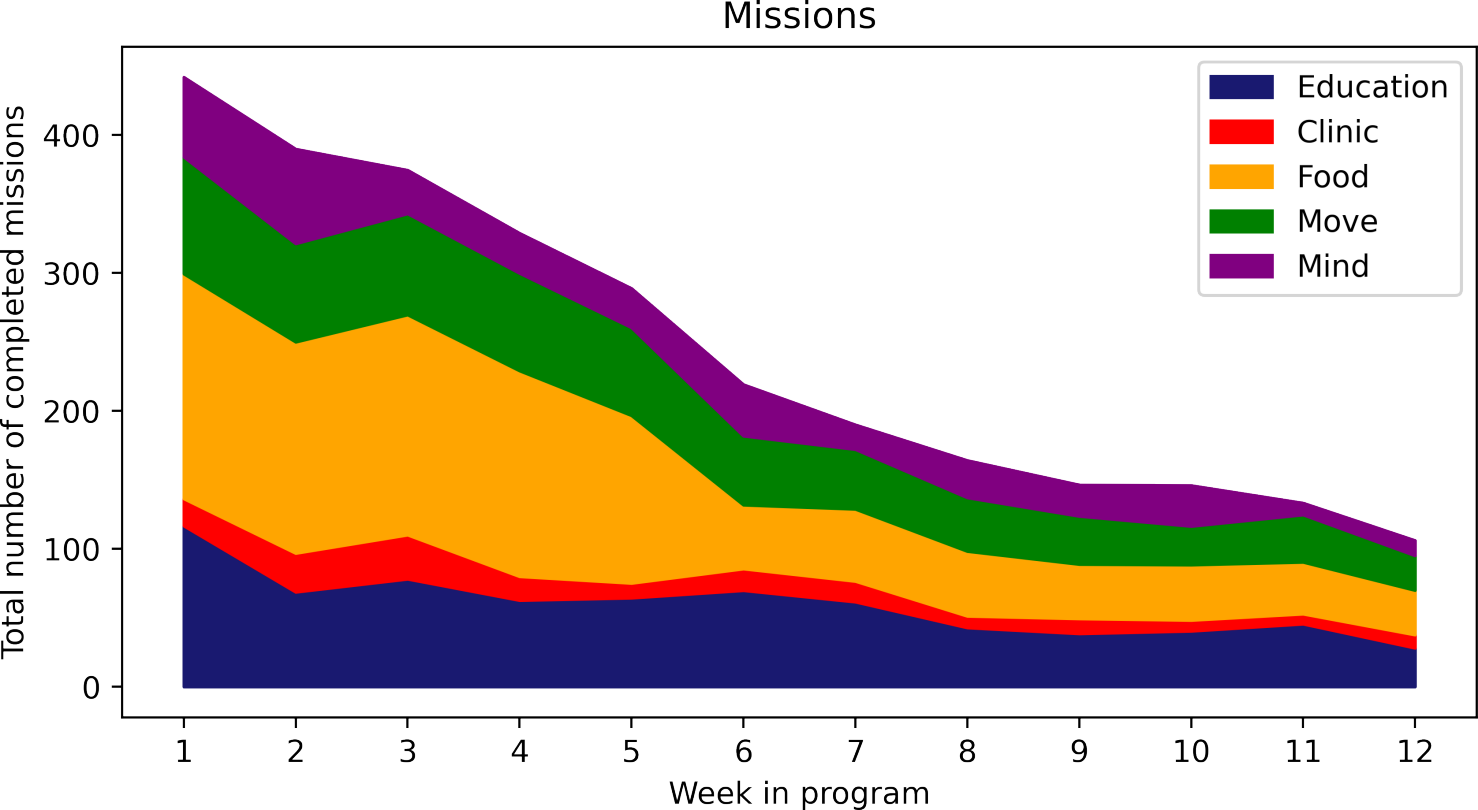
Total number of completed missions per week throughout the 12-week program, categorized by mission type.

The data showed that the participants completed, on average, 375 (SD 277.59) missions over the duration of 12 weeks. Aggregating the completed missions by participant and by day shows that the participants completed an average of 8 (SD 5.1) missions per day, not accounting for days when they did not use the app at all. However, engagement with the DTx application decreased throughout the period of 12 weeks. Figure 3 shows how the average engagement of all participants decreases with each day of the program. The different colors indicate that engagement varies between different types of missions. It is evident that the food missions were used frequently at the beginning, but decreased over time, while the education missions remained stable throughout the entire study period.

We can further split down the mission categories into specific missions. For example, both the food logger and food journal belong to the food mission category. Table 4 provides an overview of the most completed missions. The missions most often completed by the participants were logging food intake, reaching a step count goal, and watching interactive educational material. Comparing missions from the same category shows that participants more often log their food rather than use the food journal and more often engage with the interactive educational content instead of the program videos.

**Table 4.** Ten most often completed missions.

Mission	Total completions, n
Food logger	4859
Move tracking	4060
Interactive education	2452
Food journal	2211
Self-reflection	1998
Program videos	1110
Health tracking	498
Medication	485
Surveys	317
Physical activity	276

### Association Rule Mining

#### Frequent Item Sets

Generating frequent item sets from the dataset led to 81 sets, which have a minimum support of 0.1 and contain at least 2 elements. Table 5 shows 5 exemplary identified frequent item sets. The item sets show that awakenings, prolonged awake time in bed, sleep duration, sending coach messages, and stress show co-occurrences with missions in the DTx application. Particularly, low awakenings, which refer to no awakenings during the night, frequently co-occur with the education and move missions. Furthermore, decreased sleep duration, prolonged awake time in bed after waking up, sending coach messages, and increased stress frequently co-occur with different types of missions. The full list of frequent item sets can be found in Multimedia Appendix 2.

**Table 5.** Identified frequent item sets.

Frequent item set	Support	Items, n
[Awakenings↓, education, move]	0.23	3
[Sleep duration↑, education]	0.21	2
[Awake in bed↓, food]	0.19	2
[Coach message, education]	0.15	2
[Stress↑, move]	0.12	2

#### Association Rules

In total, the Apriori algorithm generated 183 rules with a minimum confidence of 0.5. We then filtered the rules based on their lift to indicate how significant the rules are in comparison to random occurrence. Only rules with a lift higher than 1.5 were included, which means that these items occur 1.5 times more often than they would by random chance, resulting in 82 rules. We then applied the chi-square test, which further validated the significance of the rules as compared to random chance and excluded 11 rules with a *P* value higher than .05. Many rules appeared multiple times, switching the position of the items from antecedent to consequent and vice versa. Therefore, no strong direction of the association was found.

Most rules show co-occurrence between different types of missions, which is natural, as a person who completes 1 type of mission is likely to complete another type of mission. The strongest support can be seen for the food, move, and education missions. To analyze the co-occurrence of items from different sources within this multimodal dataset, we only consider rules that include at least 1 item from the sleep diary and smartwatch tracking. The resulting 47 rules indicate that different missions co-occur with changes in sleep and behavior. These rules show that the missions co-occur with items derived from the digital sleep diary and smartwatch tracking. Particularly, the food, move, and education missions frequently appear in the generated rules. Regarding the sleep changes, a low number of awakenings, prolonged awake time in the morning, and improved sleep quality appear in several rules. Table 6 shows 6 exemplary rules, including their support, confidence, and lift. The full set of rules can be seen in Multimedia Appendix 3.

**Table 6.** Identified association rules.

Antecedents	Consequents	Support	Confidence	Lift
[Food, screen time↑]	[Education, move]	0.1	0.88	1.68
[Awake in bed↓, move]	[Food, education]	0.17	0.79	1.64
[Sleep quality↑, education]	[Food, move]	0.12	0.74	1.62
[Education, coach message]	[Food, move]	0.11	0.72	1.58
[Mind, awakenings↓]	[Education]	0.11	0.96	1.57
[Stress↑, move]	[Education]	0.11	0.95	1.54

**Figure 4.**
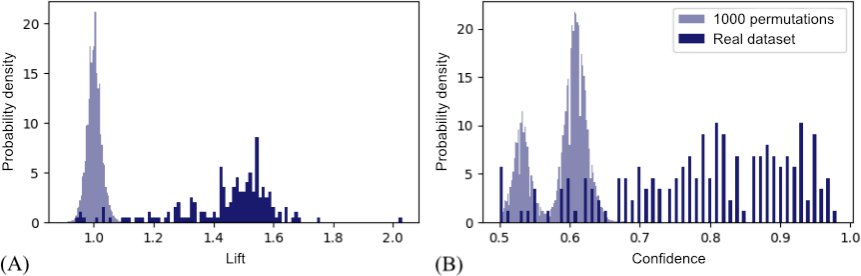
Distribution of lift (A) and confidence (B) across all rules in 1000 permutations compared with the distribution of rules generated from the real dataset.

Comparing the generated rules to rules derived from 1000 randomly shuffled permutations of the dataset, there is a clear difference in the distribution of confidence and lift in the sets of rules, as can be seen in Figure 4. This shows that the generated rules are stronger than random chance.

## Discussion

### Key Findings

The analysis of general engagement and engagement with specific missions showed overall high engagement with the DTx application. These results align with existing research suggesting that engagement plays a critical role in the success of DTx applications [24]. A key finding of this paper is the value of combining multiple data sources to create a comprehensive picture of the study. The uniqueness of this study became clear when analyzing the engagement data with the DTx application. This data stream provides key information on whether the participant actively follows the lifestyle intervention and, even more granularly, how the participant changes their lifestyle.

There are some likely explanations for the observed decline in the average number of missions completed over time. First, it is common for users to engage more actively at the start of a program as they explore the app, familiarize themselves with its features, and discover which missions are most relevant to them. This initial engagement effect often decreases as users settle into a routine. Second, users tend to complete the missions they are served on a given day, and the number of available missions decreases over time. On average, users received 38 missions per week in the first 3 weeks, compared to 28 missions per week in the final 3 weeks. When multiplied by the number of participants, this structural difference alone can account for a significant decline in total mission completions. Third, users were also required to fill out a separate diary and use another app, which may have contributed to study fatigue as the program progressed [46]. Finally, retention data show a gradual drop in active users, which would naturally result in fewer missions being completed over time [46].

The observed differences in engagement raise broader questions about the factors driving user engagement. However, to truly understand why users select certain missions over others, it is essential to consider behavior theories that explain intrinsic motivation. For example, the self-determination theory proposes that feelings of autonomy, competence, and relatedness foster intrinsic motivation to adopt health-promoting behaviors [47]. Integrating these theoretical insights with data-driven findings can inform the design of more effective DTx interventions for sustainable lifestyle changes. By applying association rule mining, the case study then provided insights into how DTx can be leveraged to promote healthier sleep habits. Association rule mining revealed that specific combinations, such as food-related and educational missions, often co-occur with changes in sleep or behavior. When comparing our identified rules with existing research, we can observe similarities. The results of Alslaity et al show similar value ranges for lift and confidence when they generate rules from the entire dataset and similar rules with switched antecedents and consequents [34]. They identify co-occurrences of users logging sleep as an activity and logging other activities such as relaxation, music, food, friends, and home [34]. However, our work dives deeper into sleep by differentiating between specific characteristics of sleep.

### Practical Implications

The implications derived from these results can be, on the one hand, useful for clinical practice and, on the other hand, useful for the developers of these digital interventions. From a clinical perspective, this paper implies that engagement with the app decreases over time. A potential research direction could be to connect the DTx application with offline coaching or physical exercises to also reach patients with OSA who do not engage with the app. This way, these treatment paths could complement each other.

From a technical perspective, this study implies that the combination of the DTx application, continuous sleep tracking, and self-reports from the digital diary offers valuable information for evaluating the application. The transaction-based format is a useful way to structure the data, which is otherwise difficult to merge in a classical table format due to its three-dimensionality, considering participants, days, and features. The identified associations are exploratory and intended to identify engagement patterns rather than to establish clinical efficacy.

### Limitations and Future Research

Our work has several limitations. First, when preprocessing the data for association rule mining—that is, the simplification of numerical data into categorical data—some richness of the data is lost. Additionally, the generic binning of features neglects individual differences between the participants. For example, more than 8 hours of sleep may be a high sleep duration for many individuals but may be a low or average sleep duration for others. Therefore, applying personalized binning by including the distribution of values within individuals would be a good research direction for future work. Second, our research was limited by knowledge about the parts of the DTx application that are not quantifiable. Features such as storytelling, design, or gamification have not been assessed in the data extracted from user interaction and may be a subject for future qualitative research on how users experienced the application. Furthermore, engagement could only be captured by the number of completed missions. Any other form of engagement—for example, the duration spent on each mission—was not captured.

Third, the clinical validity of association rules is a further limitation of the study. The association rules are able to identify co-occurrences but do not imply causation. Therefore, the underlying cause-and-effect relationship remains unclear. The metrics support, confidence, and lift can evaluate the reliability of these rules within this particular dataset, but there is little research on how to translate these insights into practice. The identified rules suggest further potential for applying this method to other longitudinal studies with multiple data sources. A possible future direction is sequential pattern mining, as the transactions have a temporal relationship that is not considered in association rule mining.

### Conclusions

The different missions within the application showed that food, movement, and education were the most actively used features of the application and showed co-occurrence with awakenings, being awake in bed in the morning, stress, and sleep duration. The paper proposes association rules on a longitudinal dataset as a possible way to evaluate digital intervention studies and shows the importance of combining multiple data sources to gain a comprehensive understanding of the participants and their behavior. Even though we cannot assume causality between the different forms of engagement and sleep patterns, association rules serve as a suitable tool to explore their relationship.

## Supplementary material

10.2196/75358 Multimedia Appendix 1 Replication code and synthetic dataset.

10.2196/75358 Multimedia Appendix 2 List of frequent item sets.

10.2196/75358 Multimedia Appendix 3 Full set of rules.

## Abbreviations

AHI: Apnea-Hypopnea Index
DTx: digital therapeutics
OSA: obstructive sleep apnea
PSG: polysomnography
RQ: research question
