# Supplementary material for: Mining Association Rules From a Multimodal Dataset of a Digital Therapeutics Application for Sleep Improvement Through a Healthy Lifestyle: Quantitative Study
Source: JMIR Form Res. 2026 Jul 6;10:e75358. doi: 10.2196/75358 (PMC13335750; doi:10.2196/75358)
Supplement: Multimedia Appendix 2 [file formative-v10-e75358-s002.docx]

| **Frequent Item Sets** | **Support** |
| --- | --- |
| [Education, Awakenings↓] | 0.26 |
| [Move, Awakenings↓] | 0.25 |
| [Education, Move, Awakenings↓] | 0.23 |
| [Food, Awakenings↓] | 0.23 |
| [Awake in Bed↓, Education] | 0.22 |
| [Awake in Bed↓, Move] | 0.22 |
| [Sleep Duration↑, Education] | 0.21 |
| [Food, Education, Awakenings↓] | 0.21 |
| [Sleep Duration↑, Move] | 0.2 |
| [Awake in Bed↓, Education, Move] | 0.2 |
| [Food, Move, Awakenings↓] | 0.2 |
| [Food, Awake in Bed↓] | 0.19 |
| [Food, Awake in Bed↓, Education] | 0.19 |
| [Food, Education, Move, Awakenings↓] | 0.19 |
| [Sleep Duration↑, Food] | 0.18 |
| [Sleep Duration↑, Education, Move] | 0.18 |
| [Food, Awake in Bed↓, Move] | 0.18 |
| [Food, Awake in Bed↓, Education, Move] | 0.17 |
| [Sleep Duration↑, Education, Food] | 0.17 |
| [Sleep Quality↑, Education] | 0.17 |
| [Sleep Quality↑, Move] | 0.16 |
| [Sleep Duration↑, Move, Food] | 0.16 |
| [Sleep Quality↑, Education, Move] | 0.15 |
| [Education, Coach Message] | 0.15 |
| [Move, Coach Message] | 0.15 |
| [Sleep Duration↑, Education, Move, Food] | 0.15 |
| [Awake in Bed↓, Awakenings↓] | 0.14 |
| [Sleep Quality↑, Food] | 0.14 |
| [Sleep Quality↑, Education, Food] | 0.13 |
| [Move, Screen Time↑] | 0.13 |
| [Food, Coach Message] | 0.13 |
| [Education, Move, Coach Message] | 0.13 |
| [Education, Screen Time↑] | 0.13 |
| [Sleep Quality↑, Move, Food] | 0.13 |
| [Sleep Quality↑, Education, Move, Food] | 0.12 |
| [Education, Move, Screen Time↑] | 0.12 |
| [Food, Education, Coach Message] | 0.12 |
| [Sleep Quality↑, Awake in Bed↓] | 0.12 |
| [Stress↑, Move] | 0.12 |
| [Pain↑, Move] | 0.12 |
| [Sleep Duration↑, Awakenings↓] | 0.12 |
| [Food, Screen Time↑] | 0.12 |
| [Mind, Awakenings↓] | 0.11 |
| [Pain↑, Education, Move] | 0.11 |
| [Stress↑, Education, Move] | 0.11 |
| [Food, Move, Coach Message] | 0.11 |
| [Awake in Bed↓, Education, Awakenings↓] | 0.11 |
| [Sleep Quality↑, Awakenings↓] | 0.11 |
| [Mind, Awake in Bed↓] | 0.11 |
| [Awake in Bed↓, Move, Awakenings↓] | 0.11 |
| [Mind, Education, Awakenings↓] | 0.11 |
| [Food, Education, Screen Time↑] | 0.11 |
| [Food, Move, Screen Time↑] | 0.11 |
| [Mind, Awake in Bed↓, Education] | 0.11 |
| [Food, Education, Move, Coach Message] | 0.11 |
| [Food, Pain↑] | 0.1 |
| [Awake in Bed↓, Education, Move, Awakenings↓] | 0.1 |
| [Food, Education, Move, Screen Time↑] | 0.1 |
| [Stress↑, Food] | 0.1 |
| [Mind, Move, Awakenings↓] | 0.1 |
