# Supplementary material for: Mining Association Rules From a Multimodal Dataset of a Digital Therapeutics Application for Sleep Improvement Through a Healthy Lifestyle: Quantitative Study
Source: JMIR Form Res. 2026 Jul 6;10:e75358. doi: 10.2196/75358 (PMC13335750; doi:10.2196/75358)
Supplement: Multimedia Appendix 3 [file formative-v10-e75358-s003.docx]

| **Antecedents** | **Consequents** | **Support** | **Confidence** | **Lift** |
| --- | --- | --- | --- | --- |
| [Sleep Quality↑] | [Awake in Bed↓] | 0.12 | 0.59 | 2.03 |
| [Education, Screen Time↑] | [Food, Move] | 0.1 | 0.79 | 1.75 |
| [Awake in Bed↓, Education] | [Food, Move] | 0.17 | 0.77 | 1.69 |
| [Food, Screen Time↑] | [Education, Move] | 0.1 | 0.88 | 1.68 |
| [Food, Awake in Bed↓] | [Education, Move] | 0.17 | 0.88 | 1.67 |
| [Awake in Bed↓, Move] | [Food, Education] | 0.17 | 0.79 | 1.64 |
| [Move, Screen Time↑] | [Food, Education] | 0.1 | 0.78 | 1.62 |
| [Sleep Quality↑, Education] | [Food, Move] | 0.12 | 0.74 | 1.62 |
| [Education, Awakenings↓] | [Food, Move] | 0.19 | 0.73 | 1.60 |
| [Mind, Awake in Bed↓] | [Education] | 0.11 | 0.98 | 1.59 |
| [Education, Move, Screen Time↑] | [Food] | 0.1 | 0.84 | 1.59 |
| [Awake in Bed↓, Education, Move] | [Food] | 0.17 | 0.84 | 1.59 |
| [Education, Screen Time↑] | [Food] | 0.11 | 0.84 | 1.58 |
| [Awake in Bed↓, Education] | [Food] | 0.19 | 0.84 | 1.58 |
| [Education, Coach Message] | [Food, Move] | 0.11 | 0.72 | 1.58 |
| [Sleep Quality↑, Move] | [Food, Education] | 0.12 | 0.76 | 1.58 |
| [Mind, Awakenings↓] | [Education] | 0.11 | 0.96 | 1.57 |
| [Food, Education, Screen Time↑] | [Move] | 0.1 | 0.95 | 1.57 |
| [Food, Awakenings↓] | [Education, Move] | 0.19 | 0.82 | 1.57 |
| [Food, Awake in Bed↓, Move] | [Education] | 0.17 | 0.97 | 1.57 |
| [Education, Screen Time↑] | [Move] | 0.12 | 0.94 | 1.56 |
| [Food, Awake in Bed↓] | [Education] | 0.19 | 0.95 | 1.56 |
| [Education, Move, Coach Message] | [Food] | 0.11 | 0.83 | 1.56 |
| [Food, Screen Time↑] | [Move] | 0.11 | 0.93 | 1.55 |
| [Move, Pain↑] | [Education] | 0.11 | 0.95 | 1.55 |
| [Move, Screen Time↑] | [Food] | 0.11 | 0.82 | 1.54 |
| [Stress↑, Move] | [Education] | 0.11 | 0.95 | 1.54 |
| [Food, Move, Screen Time↑] | [Education] | 0.1 | 0.95 | 1.54 |
| [Sleep Duration↑, Education, Move] | [Food] | 0.15 | 0.82 | 1.54 |
| [Sleep Duration↑, Move, Food] | [Education] | 0.15 | 0.94 | 1.54 |
| [Sleep Duration↑, Food] | [Education, Move] | 0.15 | 0.81 | 1.54 |
| [Food, Move, Coach Message] | [Education] | 0.11 | 0.95 | 1.54 |
| [Food, Coach Message] | [Education, Move] | 0.11 | 0.81 | 1.54 |
| [Education, Coach Message] | [Food] | 0.12 | 0.81 | 1.53 |
| [Awake in Bed↓, Move] | [Food] | 0.18 | 0.81 | 1.53 |
| [Education, Move, Awakenings↓] | [Food] | 0.19 | 0.82 | 1.53 |
| [Move, Awakenings↓] | [Food, Education] | 0.19 | 0.73 | 1.53 |
| [Food, Screen Time↑] | [Education] | 0.11 | 0.93 | 1.52 |
| [Education, Awakenings↓] | [Food] | 0.21 | 0.81 | 1.52 |
| [Food, Move, Awakenings↓] | [Education] | 0.19 | 0.93 | 1.52 |
| [Food, Awake in Bed↓, Education] | [Move] | 0.17 | 0.92 | 1.52 |
| [Pain↑, Education] | [Move] | 0.11 | 0.91 | 1.51 |
| [Sleep Duration↑, Move] | [Food, Education] | 0.15 | 0.72 | 1.51 |
| [Sleep Duration↑, Education] | [Food, Move] | 0.15 | 0.69 | 1.51 |
| [Sleep Duration↑, Food] | [Education] | 0.17 | 0.92 | 1.50 |
| [Food, Awake in Bed↓] | [Move] | 0.18 | 0.91 | 1.50 |
| [Move, Coach Message] | [Food, Education] | 0.11 | 0.72 | 1.50 |
